# Obstetrics

**Published:** 1896-06

**Authors:** Walter C. Swayne


### OBSTETRICS.

The condition which has been described under the various names of **Deciduoma malignum,** sarcoma deciduo-cellulare, and decidual carcinoma has of late attracted great attention. The condition appears to have been first described by Sänger,[1] by whom it was given the now commonly accepted name of deciduoma malignum. A large number of cases are now on record, but with two exceptions none appear to have been met with in England, the great majority occurring on the continent of Europe. The chief characteristics may be briefly stated as follows: A pathological condition following expulsion of the products of conception, marked by rapid development of new growth in the uterus, generally, it is supposed, at the placental site, producing uterine hemorrhages, fœtid discharge, enlargement of the uterus, and metastatic deposits in other organs. Its clinical characters are chiefly: a close relation to pregnancy, especially to molar pregnancy; occurrence of hemorrhages and foul discharges, owing to the formation of a friable growth resembling placental *débris*; rapid deterioration of health; and, finally, the formation of metastases. Practical lessons are, that (1) Feeble measures such as the frequent use of the tampon and curette, except of the latter for diagnostic and exploratory purposes, are not to be recommended. (2) That total extirpation of the uterus as soon as the malignant nature of the growth is established offers the best chance of recovery.

In a paper on hydatid mole, Marchand[2] states that all the forms of cells described as large nuclear decidual cells, large sarcoma cells with nuclear giant-cells derived from connective tissue, proliferating endothelial cells, and transition of unstriped muscle elements into sarcoma cells, can be recognised without difficulty in the epithelial migratory cells of the hydatid mole.

Both the original growth and the metastases contain cells corresponding to those found in the uterine decidua, and, moreover, structures the cells of which resemble in their arrangement that of the cells of the chorionic villi: the disease, if treated expectantly, seems to run a rapid course to a fatal termination. There is considerable difference of opinion as to the particular class of malignant disease under which this condition should be placed: some observers name it cellular sarcoma, others are of opinion that it is of epithelial nature. The minute pathology varies apparently, not all the cases with very similar clinical characteristics exhibiting the same pathological structures,

[1] *Centralbl. f. Gynäk.*, 1889, xiii. 132, & *Arch. f. Gynaek.*, 1893, xliv. 89, quoted by Whitridge Williams, *Tr. Am. Gynec. Soc.*, 1895, xx. 348, and in *Med. Chron.*, 1896, N.S. iv. 207.

[2] *Ztschr. f. Geburtsh. u. Gynäk.*, 1895, xxxii. 405; abstract in *Med. Chron.*, 1896, N.S. iv. 288.

while the exciting cause may be—according to observers quoted by Whitridge Williams, and in the *Medical Chronicle*, January, 1896—ordinary pregnancy (with abortion[1] or delivery at term[2]), tubal pregnancy,[3] placental polypus,[4] or hydatid mole.[5] Of twenty-six cases recorded by Sänger[6] the majority were secondary to hydatid mole, and in the metastatic deposits were found cells and structures resembling exactly those found in the villi of the hydatid mole.

That malignant growth should be taken on by the molar villi is not altogether surprising when we remember its rapid growth, the way it penetrates into the uterine mucosa, and even perforates by growth (not by pressure) the wall of the uterus. It is obvious that the rapid cell-formation of such a structure must lend itself readily to diversion into a channel leading to malignant action.

That in a lesser degree malignant growth should be taken on by retained fœtal structures is also easily understood when one considers the rapid cell-growth and edacious character of those layers of the ovum in direct contact with maternal structures.

The question of the place of this affection among malignant growths seems difficult to settle. Among the epithelial malignant tumours would appear the most rational in the majority of cases, since the greatest activity is manifested by the epiblastic elements of the segmental layers of the ovum. The connective tissue elements of the decidua which, according to Eden[7], have absorptive or edacious powers, are those of the stroma of the maternal decidua ; their activity is not apparently persistent.

Marchand's[8] conclusions on his two cases are interesting. He concludes that (1) All the cases are essentially of the same nature, although, owing to the parts involved and special circumstances, they may present individual differences. (2) All the tumours are epithelial, the layers of tissue combining in their formation being (*a*) the syncytium or uterine epithelial layer of chorion ; (*b*) the elements of the ectodermic epithelium of the chorion. (3) These elements of two orders constitute the normal portion of the serotina. (4) The derivatives from the syncytium take different forms: (*a*) large cellules

[1] Sänger, *Centralbl. f. Gynäk.*, 1889, xiii. 132. Kuppenheim, *Centralbl. f. Gynäk.*, 1895, xix. 916.

[2] Whitridge Williams, *Am. J. Obst.*, 1894, xxix. 721.

[3] Marchand, *Monatschr. f. Geburtsh. u. Gynaek.*, 1895, i.

[4] Schmorl, *Centralbl. f. Gynäk.*, 1893, xvii. 169.

[5] Pfeiffer, *Prag. med. Wchnschr.*, 1890, xv. 327. Fränkel, *Arch. f. Gynaek*, 1895, xlviii. 80. Bacon, *Am. J. Obst.*, 1895, xxxi. 679. Menge, *Ztschr. f. Geburtsh. u. Gynäk.*, 1894, xxx. 323. Löhlein, *Centralbl. f. Gynäk.*, 1893, xvii. 297. Klien, *Arch. f. Gynaek.*, 1894, xlvii. 243, 705. Nové-Josserand and Lacroix, *Ann. de gynéc. et d'obst.*, 1894, xli. 109, 216, 317. Tannen, *Arch. f. Gynaek.*, 1895, xlix. 94.

[6] *Centralbl. f. Gynäk.*, 1889, xiii. 132.

[7] *Tr. Obst. Soc. Lond.*, 1896, xxxvii. 205.

[8] *Monatschr. f. Geburtsh. u. Gynaek.*, 1895, i; abstract in *Med. Chron.*, 1896, N.S. iv. 210.

with nuclei rich in chromatin; (*b*) protoplasmic masses with multiple nuclei; (*c*) trabecular and retiform tissue, the elements of which are multi-nucleated. (5) The elements of the cellular layer often present the appearance of clear cells rich in glycogen; these cells vary in colour, but are usually smaller than those of the syncytium. (6) Molar pregnancy favours the production of malignant neoplasms. (7) The decidual cellules, properly so-called, do not participate in the formation of malignant neoplasms. (8) The participation of the connective tissue of the chorion has not yet been demonstrated. (9) Metastatic productions are almost invariably conveyed by the blood. Marchand considers that although, as epithelial growths, these tumours might be considered carcinomata, still, owing to their peculiar relation to the development of the ovum, he concludes the best designation to be tumours of the serotina.

Gottschalk, in a case in which he extirpated the uterus after some delay, considered that the placental villosities had undergone a process of malignant degeneration, the cellules of the serotina being infected with sarcomatous virus; the metastases contained epithelial elements of the primitive tumours.

Pathologists differ much in their opinion as to the nature of the growth in these cases, but clinically the following definite statements can be made: (1) That a form of malignant disease of the uterus hitherto unrecognised has been differentiated. (2) The presence of syncytial masses of protoplasm is a striking feature of several of these tumours, as (3) is also the similarity between these masses and the chorionic epithelium. In both, protoplasmic masses not divided into definite cells, deeply-staining nuclei, and vacuoles of varying size, either empty or with transparent contents, occur: these facts have been observed by Meyer,[1] Klebs,[2] Gottschalk,[3] Fränkel,[4] Marchand,[5] and Whitridge Williams,[6] and are quoted by the latter in the article referred to. All these have concluded that they had to deal with growths derived from chorionic epithelium.

In the cases now on record, the points which are most striking seem to be (1) The number of cases which occur in connection with hydatid mole. (2) The fairly constant succession of symptoms; *e.g.*, hemorrhages, at first, followed by fœtor of discharges and cachexia. (3) The undoubtedly malignant character of the new growth, its characteristics being rapid invasion of tissue, rapid recurrence, early necrosis, and early metastasis. (4) The entire futility of attempting to deal with this condition by palliative or, rather, minor operative measures: *e.g.*, in one case after repeated *tamponnement* death apparently

[1] *Arch. f. Gynaek.*, 1888, xxxiii. 53.
[2] *Die allgemeine Pathologie.* 1889, II. 612.
[3] *Arch. f. Gynaek.*, 1890, xxxvii. 251 & 1894, xlvi. 1. *Berl. klin. Wchnschr.*, 1893, xxx.
[4] *Loc. cit.* [5] *Loc. cit.* [6] *Loc. cit.*

from septicæmia, and in several others symptoms of septic infection, occurred as the result of measures taken for relief; the cases subjected to early vaginal hysterectomy recovered and remained well for some months.

The case described by Whitridge Williams[1] has some very peculiar and important features; one is, the relation between the development in time and amount of growth of the primary tumour and the metastasis in the vulva; the vulvar metastasis developed two weeks after labour. If the uterine tumour was primary—and from the description there is no reason to suppose the contrary—its growth was much less rapid than that of the metastasis, and it must have been in existence two weeks after delivery; the occurrence of metastasis through the vessels renders this possible, the conveyance of a thrombus being sufficiently rapid. Instances are given by Schmorl[2] of the occurrence of thrombi in pulmonary arteries composed of placental giant-cells, without any tumour of uterus or placenta. The author, Whitridge Williams, mentions thrombi of chorionic epithelium formed in venous channels of the tube-wall in tubal pregnancy; and Pestalozza,[3] after a hydatid mole had been expelled, found pulmonary thrombi composed of myxomatous chorionic villi. If we allow to the tissues forming the thrombus the very evident malignant character of the primary formation from which they are separated, the rapid metastasis seems easily explicable. Some of the clinical features are worthy of mention: most of the patients are young, fifty per cent. of the recorded cases occurring before thirty, and seventy per cent. before forty, the remainder between forty and fifty-five; this is not remarkable, seeing that the growth is directly related to pregnancy. The character of the hemorrhage, which is usually intermittent, severe, and occurring in gushes, indicates the opening of a vessel; the uterus is enlarged, but its cavity filled, the contents friable and easily removed, leaving a cavity in the wall of the uterus. The recurrence of the growth is very rapid, as are also the metastases; vaginal metastases are common. The fact of vaginal metastases occurring after a tubal pregnancy seems to enable us to preclude the possibility of their production by direct implantation.

The course of the disease is rapid and ends fatally if not interrupted by proper surgical treatment. The diagnosis presents small difficulty in the later stage when metastases have occurred, but considerable difficulty exists in coming to a correct conclusion in the early stages when the microscopic evidence is probably all that can be obtained. The presence of syncytial masses between the muscular bundles and fibres, and extending into the muscular tissue, is the characteristic of the growth, and can enable a definite opinion to be formed.

[1] *Loc. cit.*

[2] *Pathologisch-anatomische Untersuchungen über Puerperal-Eklampsie.* 1893.

[3] *Morgagni*, 1891.

The practical conclusions to be drawn from the cases reported appear to be, that in any case where hemorrhage occurs in connection with the puerperal state, or after the puerperal period, more especially in cases of hydatid mole, the necessity for exploration of the uterus after dilatation of the cervix must be remembered. The symptoms indicating the necessity for this treatment are, the character of the hemorrhage and the enlargement of the uterus. These both appear to give an indication which, in most cases, is fairly clear: the gushing character of the hemorrhage, its irregular occurrence, the increase in size of the uterus after its involution, or involution becoming stationary, being rather different to the conditions of hemorrhage and enlargement occurring in simple or benign cases.

After exploration, a careful microscopical examination of any tissue removed should be made. In reading the reports of the cases of deciduoma, one or two cases occur to one's mind in which the symptoms were such that it seems not improbable that they may have been cases of this disease. In the absence of microscopic evidence and of a history of the ultimate fate of the patient, one cannot say more than that if at the time of their occurrence the subject of this disease had received the attention it has of late, it would have led to investigations being made with a view to excluding its presence. The knowledge that this rare condition has been observed, should lead to the careful investigation of all material removed from the uterus after curetting.

The much debated questions of its minute pathology do not appear to be of very great practical importance; the necessity of early operation and radical treatment is obvious, whether the neoplasm is carcinoma or sarcoma of the previously recognised types occurring in the uterus with a relation to pregnancy wholly accidental, or a distinct form developed from and containing decidual tissues.

At the April meeting of the Obstetrical Society of London,[1] two cases of deciduoma malignum were described—one by Mr. Alban Doran for Mr. Rutherford Morison, and one by Dr. Herbert Spencer. Dr. Eden also read a paper on the subject. Dr. Spencer's case occurred twenty-eight days after a normal labour, and ran the course described in other cases. After death an ulcerated and gangrenous growth was found at the placental site, the ulceration having nearly perforated the fundus. Secondary deposits were found in the cervix and lungs, and the character of the growth and secondary deposits was the same, being apparently a large-celled sarcoma with typical syncytium. Dr. Eden in his paper criticised the conclusions of other observers. He considered that the origin of the growth in the decidua was not proved, and that plasmodial masses

[1] *Brit. M. J.*, 1896, i. 976.

called syncytial are found in ordinary sarcoma of other parts of the body. He considered that Whitridge Williams's case was an ordinary sarcoma, and that there was no evidence to show that the uterine growth was primary. He suggested that Marchand's case following tubal gestation was due to primary growth in the tube unconnected with pregnancy, of which he considered there was no anatomical evidence. In the discussion[1] which followed, Drs. Kanthack, Clarence Webster, and Fothergill agreed in the main with Dr. Eden's criticisms. Dr. Fothergill considered that there was no proof that fœtal epiblast would form new growths and metastases. Mr. Bland Sutton considered that deciduoma was a sarcoma arising in decidual tissue. He felt sceptical as to varieties supposed to originate in chorionic villi. Mr. Alban Doran said that Mr. Morison's case was an instance of deciduoma malignum. He considered, however, that the malady could not be admitted to be distinct from sarcoma stimulated by gestation, while two cases quoted as deciduoma in the *Centralblatt für Gynäkologie* for May 2nd were more probably epithelioma. Dr. Spencer pronounced the view that the condition was that of a malignant growth developed from the products of conception. The general tenor of the debate seemed to be: (1) that the plasmodial masses were not necessarily syncytial, but found in ordinary rapid-growing sarcoma. (2) that syncytial masses could not be identified with accuracy merely from microscopic appearances; (3) that quite possibly many of the cases were sarcoma or carcinomatous growth stimulated by or developed soon after pregnancy, but not necessarily connected with the products of conception; (4) that it was yet too soon to add a new disease to the list.

WALTER C. SWAYNE.

